# Coexistence of Intra- and Intermolecular Hydrogen Bonds: Salicylic Acid and Salicylamide and Their Thiol Counterparts

**DOI:** 10.1021/acs.jpca.0c11183

**Published:** 2021-02-16

**Authors:** Samira Gholami, Mohammad Aarabi, Sławomir J. Grabowski

**Affiliations:** † Dipartimento di Chimica Industriale, Università degli Studi di Bologna, Viale del Risorgimento 4, I-40136 Bologna, Italy; ‡ Faculty of Chemistry, University of the Basque Country and Donostia International Physics Center (DIPC), P.K. 1072, 20080 Donostia, Spain; § IKERBASQUE, Basque Foundation for Science, 48011 Bilbao, Spain

## Abstract

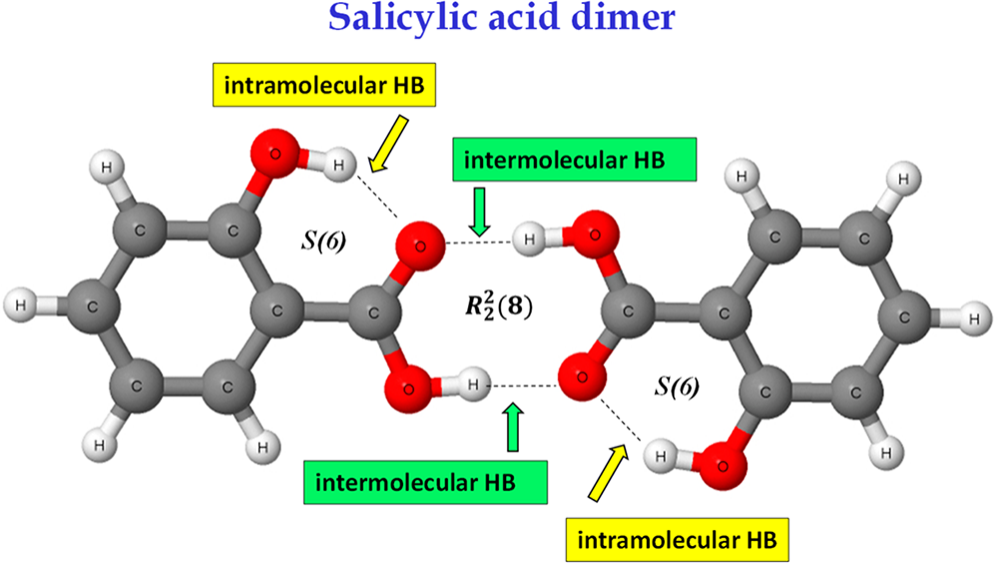

The ωB97-XD/6-311++G(d,p) calculations were carried out on dimers and monomers of salicylic acid and salicylamide as well as on their thiol counterparts; different conformations of these species were considered. The searches through the Cambridge Structural Database were performed to find related structures; thus the analysis of results of these searches is presented. Various approaches were applied to analyze inter- and intramolecular hydrogen bonds occurring in the above-mentioned species: natural bond orbital (NBO) method, symmetry-adapted perturbation theory (SAPT) approach, the quantum theory of atoms in molecules (QTAIM), and the electron localization function (ELF) method. The results of calculations indicate a slight mutual influence of inter- and intramolecular hydrogen bonds. However, the frequent occurrence of both interactions in crystal structures indicates the importance of their coexistence. The occurrence of intramolecular chalcogen bonds for trans conformations of species analyzed is also discussed.

## 1 Introduction

**Scheme 1:**
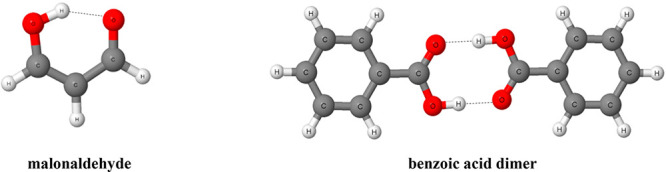
Malonaldehyde (Left) and Dimer of Benzoic Acid (Right) Broken lines indicate hydrogen bonds.

There are structural fragments that are often observed in various crystal structures.^1,2^ For example, it concerns motifs containing hydrogen bonds.^3−5^ In an early study Etter presented rules of a formation of hydrogen bonds in structures of organic compounds;^6^ among general rules there is a statement that “six-membered-ring intramolecular hydrogen bonds form in preference to intermolecular hydrogen bonds.” Such rings closed by hydrogen bonds are designated as the *S*(6) motifs in the graph-set assignments.^6^ The latter designation indicates that six atoms in the ring are connected by five covalent bonds and one hydrogen bond; such an arrangement occurs in the molecular structure of one of the conformers of malonaldehyde (Scheme 1) where the −O–H···O=C–C=C– ring is observed. It has also been pointed out that most of carboxylic acid dimers as well as numerous dimers of amides match the *R*_2_^2^(8)motif.^6^ The latter arrangement is built up of eight atoms, and it contains two proton donors and two proton acceptors (marked by the subscript and the superscript, respectively). For example, this motif is observed in the structure of benzoic acid dimer (Scheme 1) where two carboxylic groups are linked by two O–H···O hydrogen bonds; it means that for eight atoms six covalent bonds and two hydrogen bonds occur; i.e., −C=O···H–O–C=O···H–O– sequence is observed.

The structures presented in Scheme 1 are often classified as those containing resonance assisted hydrogen bonds (RAHBs).^7,8^ In malonaldehyde and in numerous related species the intramolecular RAHBs that occur are accompanied by systems of conjugated single and double bonds. The following characteristics are observed for such systems; the π-electron delocalization in the six-membered ring, the increase of the polarization of the O–H proton donating bond, the movement of the H atom to the center of the O···O distance, and consequently the increase of the strength of the hydrogen bond.^7,8^ The similar electron charge delocalization is often observed for intermolecular RAHBs like in dimers of carboxylic acids where the *R*_2_^2^(8) motifs occur. This delocalization leads to the equalization of formal C–O and C=O bonds in carboxylic groups and consequently to the increase of the strength of hydrogen bonds.^9^ It is worth noting that this equalization may be a complex process since it may also be related to the mesomeric effect in the carboxylic groups^10^ or to the disorder phenomenon.^11^ Such disorder was analyzed in the crystal structure of benzoic acid, for example.^12^ The concept of RAHB systems is negated in several studies since it was found that similar systems to those possessing conjugated single and double bonds but containing a skeleton of single bonds are characterized by hydrogen bonds of a similar strength.^13^ It was also justified that for malonaldehyde, as well as for formic and acetic acid dimers, the resonance forms that are indicative of RAHB^7,8^ do not occur.^14,15^ However, various studies indicate that in spite of the questioned RAHB concept, the π-electron delocalization occurs for systems usually classified as the resonance assisted ones that is connected with the increase of the strength of the hydrogen bond.^9,16,17^ In numerous studies, the RAHB term is used for systems where π-electron delocalization occurs. That is also in this study, in spite of doubts and controversies concerning the RAHB concept.

It seems that dimers of carboxylic acids are the most often occurring intermolecular RAHBs since other systems linked by such interactions as, for example, amide dimers, amide–amidine couplings, thiocarboxylic acid dimers, or DNA base pairs^7^ are not so common. The Cambridge Structural Database (CSD)^18,19^ searches were performed in one of the recent studies,^20^ and 4722 cases of carboxylic acid dimers linked by two O–H···O hydrogen bonds occur, while the analogues search for thiocarboxylic acids leads to three dimers; both searches were performed for conditions to find accurate crystal structures without errors. The search with the same conditions performed to find dithiocarboxylic acids, i.e., structures containing the −CS_2_H group, led to the only one structure, the crystal structure of trithiocarbonic acid.^20^

The aim of this study is to analyze o-substituted derivatives of carboxylic acids, particularly the interrelation between intermolecular and intramolecular hydrogen bonds. In dimers of these species linked by carboxylic groups the *S*(6) and *R*_2_^2^(8) motifs described earlier here occur. The 2-hydroxybenzoic acid (salicylic acid) and salicylamide are analyzed here as well as their thiol analogues thiosalicylic acid and 2-mercaptobenzamide. These dimers are compared here with benzoic acid (Scheme 1) and benzamide dimers where only intermolecular hydrogen bonds occur. The above-mentioned systems were the subject of various investigations. There is a great number of studies concerning benzoic acid and benzamide; however there are also numerous studies concerning the remaining above-mentioned moieties, particularly salicylic acid and salicylamide. One can mention the compression of a crystal of salicylamide from ambient pressure to 5.1 GPa.^21^ It was found that the change of pressure condition does not influence the polymorphic form since it is a monoclinic system and *I*2/*a* space group for the broad range of the pressure. However, if the salicylamide crystal is grown directly from the solution at 0.2 GPa a new phase salicylamide-II is formed corresponding to the orthorhombic system and the *P*2_1_2_1_2_1_ space group.^21^ In another study, crystal structures of monosubstituted salicylic acids were discussed.^22^ One should also mention theoretical analysis of the halosalicylic acids^23^ or of different conformations of the salicylic acid monomer and dimer.^24^ Numerous conclusions come from results of calculations of the latter study; one of the most important is that the most energetically favorable dimer structure is the one where intramolecular O–H···O hydrogen bonds are formed between hydroxyl groups and the carbonyl oxygen centers and where the centrosymmetric dimer is linked through two equivalent O–H···O intermolecular hydrogen bonds.

In this study, the above-mentioned species are discussed to present the interrelations between two structural motifs, *S*(6) and *R*_2_^2^(8), that occur most often in crystal structures. Besides, the intramolecular S–H···O hydrogen bonds are rarely subjects of analyses. Hence this study intends to fill the existing gap, at least partly. Various theoretical approaches are applied here: natural bond orbital (NBO) method,^25,26^ symmetry-adapted perturbation theory (SAPT) approach,^27^ the quantum theory of atoms in molecules (QTAIM),^28^ and the electron localization function (ELF) method.^29−32^

## 2 Computational Details

The calculations were performed with the Gaussian 16 set of codes.^33^ The cis and trans conformers for monomers and dimers of salicylic acid and salicylamide as well as for their thiol analogues thiosalicylic acid and 2-mercaptobenzamide were optimized. The same types of calculations were performed for analogues species characterized by the lack of the hydroxyl or thiol group responsible for the existence of intramolecular hydrogen bonds for cis conformers, i.e., for benzoic acid and for benzamide.

The ωB97XD functional^34^ and the Pople style 6-311++G(d,p) basis set^35^ were applied. The ωB97XD functional was used in calculations because it has been found that it produces more reliable results than other most often applied functionals.^36^ Similarly, it was found that the Pople basis set applied here gives satisfying results; “the aug-cc-pVDZ basis set generally gives less satisfactory geometries than 6-311++G**. This is probably a result of the latter being of triple-ζ quality for the valence electrons, whereas the former is of double-ζ quality.”^37^ Frequency calculations have been carried out at the same level as geometry optimizations, and it has been confirmed that optimized structures correspond to energetic minima.

The “quantum theory of atoms in molecules”, QTAIM,^28^ was also applied in this study to analyze characteristics of critical points that correspond to hydrogen bonds. The AIM2000^38^ and AIMAll^39^ programs were used to perform QTAIM calculations. The NBO method^25,26^ was used to calculate the energies of orbital–orbital interactions. For the A–H···B hydrogen bond, the n_B_ → σ_AH_* overlap is characteristic and the most important orbital–orbital interaction.^25,26^ n_B_ marks the lone electron pair of the B proton accepting center, and σ_AH_* is an antibond orbital of the Lewis acid unit A–H bond. This interaction energy is expressed by eq 1.
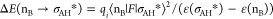
1⟨n_B_|*F*|σ_AH_*⟩ is the Fock matrix element, (ε(σ_AH_*) – ε(n_B_)) is the orbital energy difference, and *q_i_* is the donor orbital occupancy. The n_O_ → σ_OH_* and n_O_ → σ_NH_* overlaps occur for intermolecular interactions discussed here, while the n_O_ → σ_OH_* and n_O_ → σ_SH_* overlaps occur for the intramolecular hydrogen bonds.

The symmetry-adapted perturbation theory (SAPT) approach^27^ was used to deepen the understanding the nature of intermolecular interactions, and the interaction energy of two closed-shell moieties is obtained in this approach directly as a sum of defined energy terms. The Psi4 program was applied to carry out the corresponding calculations.^40^ The SAPT2 calculations were performed with the 6-311++G(d,p) basis set, i.e., SAPT2/6-311++G(d,p), for the structures optimized at the ωB97XD/6-311++G(d,p) level. The SAPT interaction energy is the sum of the following contributions: the first-order electrostatics (*E*_elst_^(1)^), second-order induction (*E*_ind_^(2)^), and dispersion (*E*_disp_^(2)^) energies and their exchange counterparts first-order exchange (*E*_exch_^(1)^), second-order exchange-induction (*E*_exch-ind_^(2)^), and exchange-dispersion (*E*_exch-disp_^(2)^). The main part of the energy of interaction is covered up to the second order in SAPT approach. The higher-order induction and exchange-induction energies are included in the Hartree–Fock “delta” correction term δ*E*_HF_. Thus, this second order interaction energy SAPT2, calculated here, is expressed by eq 2.
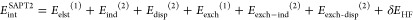
2The electron localization function (ELF) method was applied to calculate orbital occupancies in monomers and dimers analyzed and to calculate other ELF characteristics as well as to discuss the electron charge shifts resulting from complexations. The ELF calculations have been performed using TopMod program,^41^ and the ELF isosurfaces were depicted by Chimera 1.11.2 package.^42^

## 3 Results and Discussion

### 3.1 Dimers of o-Substituent Benzoic Acid and Benzamide Derivatives in Crystal Structures

The Cambridge Structural Database, CSD,^18,19^ searches (August 2020 release) have been carried out here for dimers of derivatives of benzoic acid and benzamide linked by two intermolecular hydrogen bonds as well as containing intramolecular A–H···O hydrogen bonds, where A = C, N, O, Si, P, S, in other words for the A–H proton donating bond with A designating the element of 14th, 15th, and 16th groups of the second and third rows of the periodic table. The A–H bond is situated in the ortho-position in relation to the carboxylic or amide group. The sample was fixed in such a way that there are no substituents for the remaining H atoms of the benzene ring. Hence the benzoic acid and benzamide derivatives are considered with the ortho-substituent containing A–H proton donating bond. For all searches performed here the same criteria were applied; i.e., exclude structures with unresolved errors, exclude powder structures, no polymeric structures, nondisordered structures, only single crystal structures, *R* ≤ 7.5%, esd values for CC bond lengths that are less than or equal to 0.005 Å.

**Figure 1:**
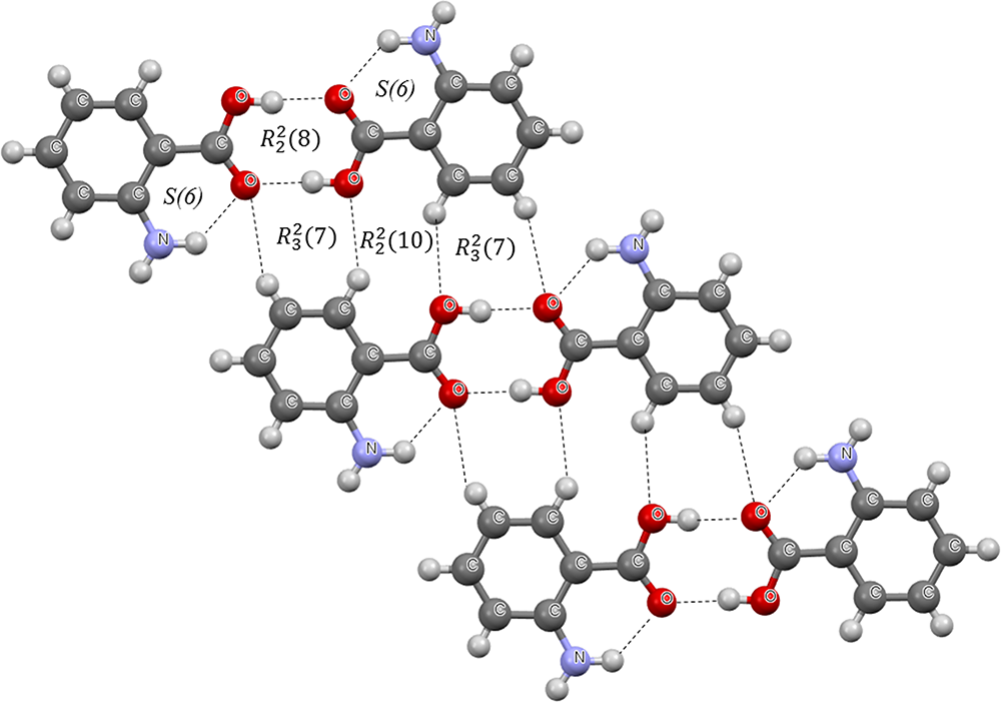
Fragment of the crystal structure of 2-aminobenzoic acid.^44^ Broken lines indicate hydrogen bonds. The designations of motifs are included.

In the case of carboxylic group attached to the benzene ring the A–H···O intramolecular hydrogen bond may be formed between the carbonyl oxygen center or the hydroxyl oxygen of this group and the A–H bond of the ortho-substituent. For the former A–H···O=C– arrangement 103 structures were found that fulfill the search conditions mentioned above; mostly these are the centrosymmetric dimers with two equivalent intermolecular O–H···O hydrogen bonds. Only for 10 structures are these bonds not equivalent since they differ in geometries. For this search, for all species, the intramolecular hydrogen bonds occur for both units of the dimers. These are mainly the N–H···O hydrogen bonds. Only in 16 dimers do the intramolecular C–H···O hydrogen bonds occur, and in 7 dimers O–H···O hydrogen bonds are observed. However, the latter case concerns different measurements of the same salicylic acid compound. The H···O intramolecular contacts that are considered here are lower than 2.4 Å; thus distances are lower by at least 0.2 Å than the corresponding sum of van der Waals radii.^43^ This distance criterion for intramolecular hydrogen bond contacts is applied for other searches performed here. Figure 1 presents fragment of the crystal structure of 2-aminobenzoic acid^44^ where the centrosymmetric dimers are observed linked by two equivalent O–H···O hydrogen bonds; the intramolecular N–H···O hydrogen bonds occur apart from the above-mentioned intermolecular O–H···O interactions. Figure 1 also shows that two dimers are linked between themselves by four weaker C–H···O hydrogen bonds; the corresponding arrangements may be classified as the *R*_3_^2^(7) and *R*_2_^2^(10) motifs according to the graph theory assignments.^6^

**Figure 2:**
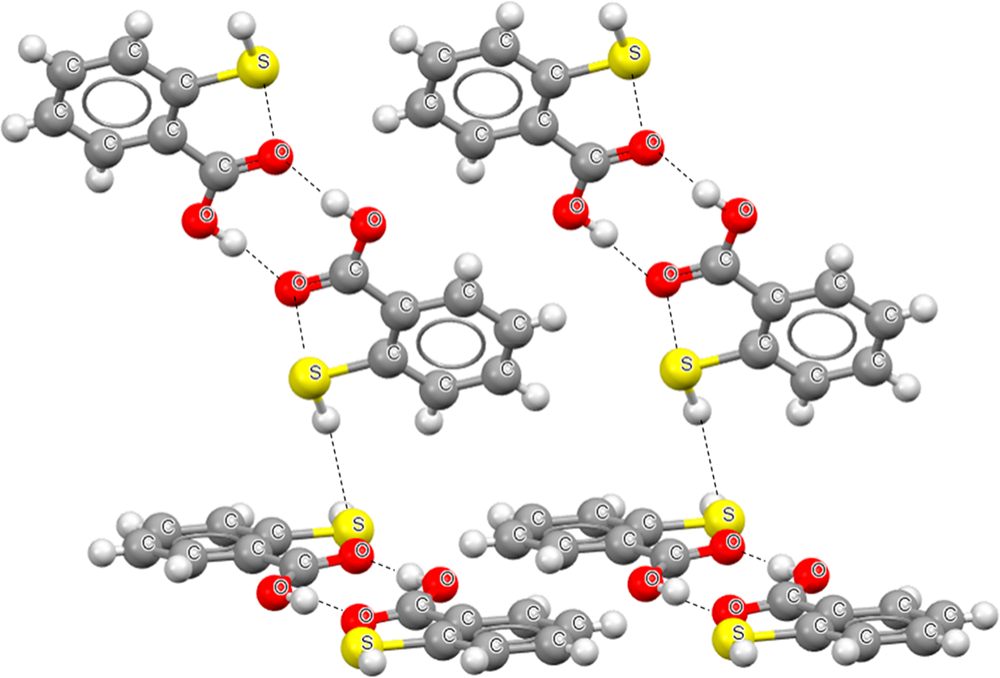
Fragment of the crystal structure of thiosalicylic acid.^45,46^ Broken lines indicate hydrogen bonds and intramolecular chalcogen bonds.

The structures with intramolecular hydrogen bonds discussed here may be classified as cis configurations. The CSD search was also performed to find the structures characterized by the occurrence of arrangements classified as the trans configurations. In such structures the intramolecular hydrogen bonds are not formed since the A–H proton donating bond is not directed toward the oxygen Lewis base center. This search led to the finding of only two entries of the same structure of thiosalicylic acid (two measurements of this structure that fulfill the search conditions mentioned above here have been stored in CSD).^45,46^Figure 2 presents the fragment of this crystal structure. The centrosymmetric dimers of thiosalicylic acid are connected through carboxylic groups by two equivalent O–H···O hydrogen bonds. These are the mentioned earlier here the *R*_2_^2^(8) motifs. Additionally each dimer is linked with another one by S–H···S hydrogen bond. The intramolecular S–H···O hydrogen bonds are not observed for this structure; however, there are the intramolecular S···O contacts that lead to the closure of the five-member rings. These interactions may be classified as the intramolecular chalcogen bonds^46^ since the S···O distance being equal to 2.762 Å is smaller than the corresponding sum of van der Waals radii, 3.25 Å.^5,43^ The cis counterpart of the thiosalicylic acid characterized by the occurrence of intramolecular hydrogen bond was not found in CSD.

The next search performed here concerns derivatives of benzoic acids with the A–H proton donating bond located in the ortho-substituent and interacting with the hydroxyl oxygen center of the carboxylic group. Only one structure, *N*-(*o*-anisole)anthranilic acid,^47^ has been found that follows the search criteria specified earlier and applied here. It is worth noting that two O–H···O intermolecular hydrogen bonds that link the carboxylic groups are not equivalent. For this structure the intramolecular N–H···O hydrogen bonds occur.

**Figure 3:**
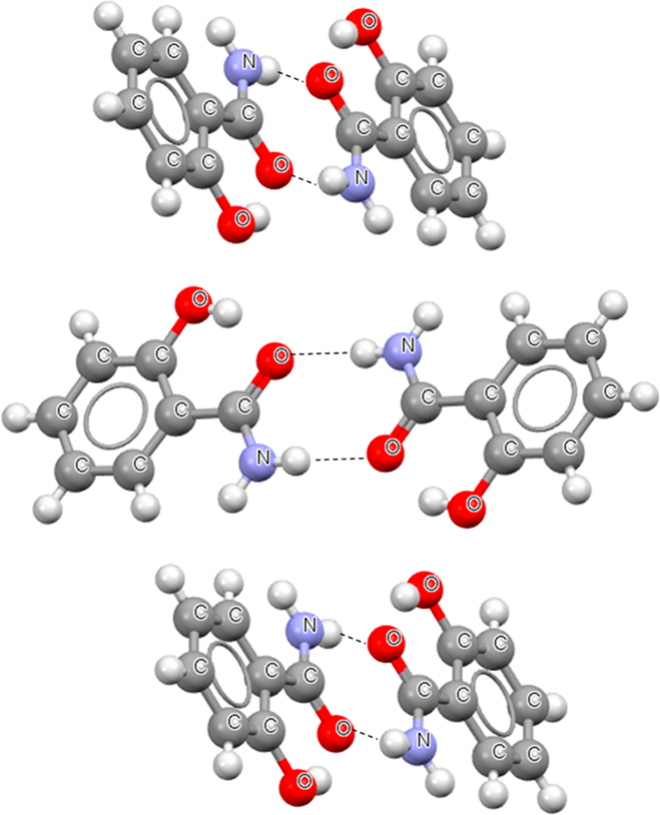
Fragment of the crystal structure of 2-hydroxybenzamide.^21^ Broken lines indicate hydrogen bonds.

The next searches concern derivatives of benzamide. Similarly, as for the derivatives of benzoic acid, the intramolecular hydrogen bonds with amide group may be formed through nitrogen or through oxygen of the C=O bond. In the case of the former arrangement, the search through CSD does not indicate any crystal structure. This may be explained by the possible repulsion between H atoms of the −NH_2_ group and the AH proton donating bond. For the hydrogen bond with carbonyl oxygen center only 17 structures are the result of the CSD search. In all structures the dimers connected by two N–H···O hydrogen bonds are observed. In 15 cases these are centrosymmetric dimers with equivalent hydrogen bonds between amide groups. In 2 cases the hydrogen bonds are not equivalent. In 10 cases of these 17 structures of amides the intramolecular N–H···O links are observed. In 7 structures these are O–H···O connections. However, the latter arrangement concerns 4 crystal structures of 2-hydroxybenzamide measured at different pressures.^21^Figure 3 presents the fragment of the crystal structure of the latter compound (SAMID03 refcode, synchrotron radiation; at 0.3 GPa and at 293 K). The search through CSD to find benzamides with o-substituents located close to the C=O bond of amide group and classified as trans conformations has not led to any results.

**Figure 4:**
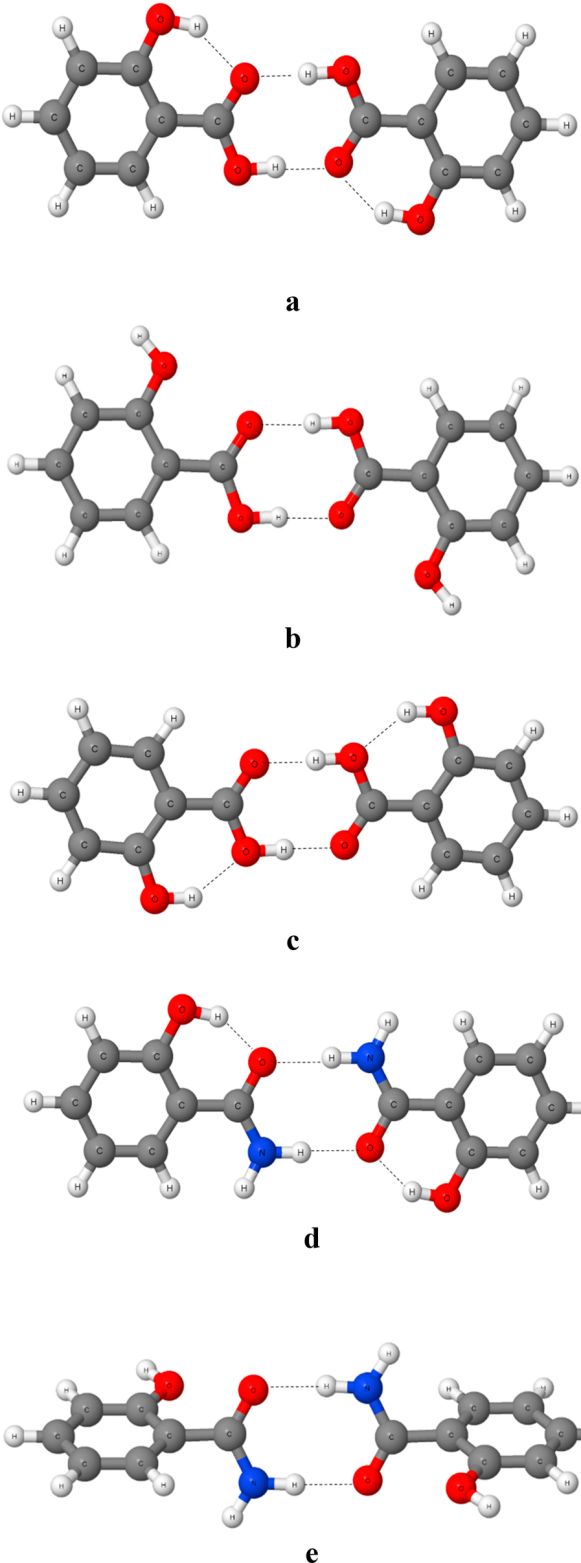
Conformations of dimers of the salicylic acid (SA) and 2-hydroxybenzamide (HBA), where broken lines indicate hydrogen bonds: (a) cis conformer of SA; (b) trans conformer of SA; (c) conformation of SA with intramolecular hydrogen bond where the hydroxyl O-center is the proton acceptor; (d) cis conformation of HBA; (e) trans conformation of HBA.

There is an interesting observation resulting from the searches performed; there are no structures with intramolecular S–H···O hydrogen bonds, neither for derivatives of benzoic acid nor for benzamide derivatives. However, it is obvious that the *R*_2_^2^(8) motifs are very common since they occur for both classes of structures. The latter arrangements are most often observed since it is partly connected with the important role of the electrostatic forces.^48^ For these species the minimum and maximum electrostatic potentials are often observed for the carbonyl oxygen and the O–H or N–H bond hydrogen, respectively. It was analyzed for the benzoic acid and its derivatives,^49^ for example. However, other issues related to the structures discussed here are still open. What is the reason that the intramolecular hydrogen bonds are formed with the carbonyl oxygen acceptor while they are rare for the hydroxyl oxygen? Why, in a case of the A–H proton donating bonds in ortho-substituents, are the cis conformations preferable to the trans ones? It is probably related to the energies of systems considered. One may refer to the ωB97-XD/6-311++G(d,p) results of calculations performed here. Figure 4 presents conformations of the salicylic acid and 2-hydroxybenzamide (salicylamide). The cis conformation of the salicylic acid dimer, treated as the reference one (Figure 4a), is characterized by lower energy than its trans conformer (Figure 4b) by 21.3 kcal/mol for dimers and by 11.1 kcal/mol for the corresponding pair of monomers. The conformation with the intramolecular hydrogen bonds formed with the hydroxyl oxygen atoms (Figure 4c) possesses the energy higher by 4.2 kcal/mol than the reference system for dimers and by 3.4 kcal/mol for monomers. The similar situation is observed for the sulfur counterparts of the salicylic acid dimer. The trans conformation of the thiosalicylic acid is higher in energy than the cis counterpart being the reference system by 1.4 kcal/mol for dimers, and the same difference in energies is observed for the corresponding monomers. The dimer with the S–H···O intramolecular hydrogen bonds with hydroxyl oxygen centers acting as the proton acceptors is higher in energy by 3.6 kcal/mol than the energy of reference dimer; for the monomers this difference amounts to 3.0 kcal/mol. For 2-hydroxybenzamide the trans conformation (Figure 4e) is higher in energy than the reference cis counterpart (Figure 4d) by 21.3 kcal/mol for dimers and by 11.0 kcal/mol for monomers. For the sulfur corresponding dimers, 2-mercaptobenzamide, the trans conformer is higher in energy by 3.7 kcal/mol than the energy of the cis system, while for monomers this energy difference is equal to 2.3 kcal/mol.

One can see that for all dimers, for carboxylic acids, and for amides with OH or SH groups in the ortho position, the most stable, i.e., possessing the lowest energies, are the dimers with the O/S–H bond close to the carbonyl oxygen and with the O/S–H···O intramolecular hydrogen bonds (cis systems). The lowest energy difference of 1.4 kcal/mol is observed for thiosalicylic acid dimer between trans and cis conformations where for both species the SH group is close to the carbonyl group. This may be the reason why the trans thiosalicylic acid structure is observed in the crystal structure discussed earlier here since such a structure is easily stabilized by the environment (Figure 2).

### 3.2 Geometry of Inter- and Intramolecular Hydrogen Bonds in Derivatives of Salicylic Acid and Salicylamide

For six-member rings closed by the resonance assisted hydrogen bonds (RAHBs) the equalization of bonds of the ring is observed.^7,8^ This equalization results from the π-electron delocalization and not from the mixture of resonance forms.^14,15^ For example, for the malonaldehyde (Scheme 1) and its derivatives and for similar conjugated systems, the equalization of the C=C and C–C pair of bonds on one hand and the C=O and C–O bonds on the other hand is observed. The similar effects occur for the intermolecular RAHBs where π-electron delocalization occurs.^7,8,50^ For example, for carboxylic acid dimers linked by two O–H···O hydrogen bonds (see benzoic acid dimer, Scheme 1) the equalization of C–O and C=O bonds is observed.^50,51^ For formamide dimer and similar systems linked by two N–H···O hydrogen bonds, the shortening of the N–C and the lengthening of the C=O bonds in amide group occur in comparison with the corresponding monomer system.^52^

**Scheme 2:**
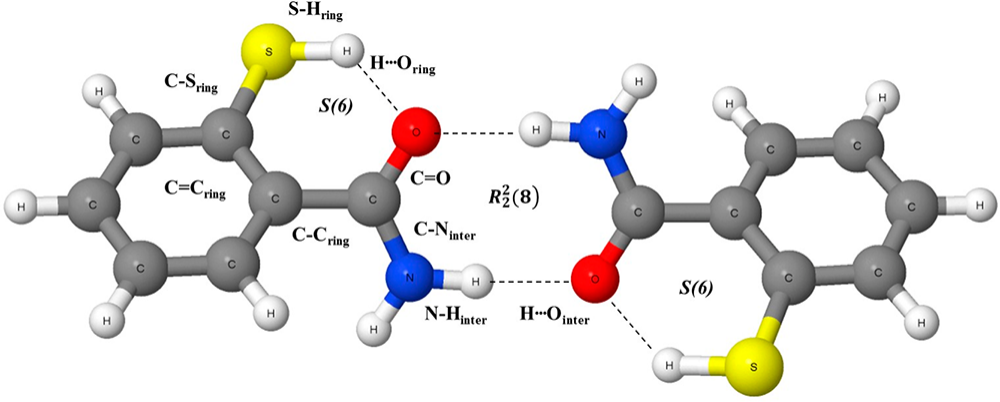
Dimer of 2-Mercaptobenzamide The designations of bonds that are used in the text and in Table 1 are shown. Broken lines indicate hydrogen bonds.

The species analyzed in this study are characterized by intra- and intermolecular hydrogen bonds, and the interrelation between these two types of interactions is discussed. The corresponding trans conformations as well as the benzoic acid and benzamide systems where intramolecular hydrogen bonds do not occur are also presented for comparison. Table 1 presents the bond lengths related to the *S*(6) and *R*_2_^2^(8) motifs that occur in monomers and dimers investigated here. For the convenience of further descriptions, the parameters of the former motif occurring in cis conformations and the corresponding parameters of the trans conformations are designated by the subscript “ring” and the parameters of the *R*_2_^2^(8) motif are marked by the subscript “inter”. The C=O bond is common for both motifs; thus it does not possess a subscript. Scheme 2 shows the dimer of 2-mercaptobenzamide to present the above-mentioned designations.

**Table 1:** Selected Bond Lengths (in Å) of the Monomers and Dimers of Benzamide and Benzoic Acid as Well as Their Simple Ortho-Substituted Derivatives^a^

compd	C–C_ring_	C=C_ring_	Δ*r*	C–O/S_ring_	C=O	C–O/N_inter_
Monomers						
benzamide	1.502	1.394	0.108		1.214	1.368
benzoic acid	1.487	1.394	0.093		1.204	1.347
salicylamide	**1.484**	**1.411**	**0.073**	**1.334**	**1.232**	**1.357**
2-mercaptobenzamide	**1.501**	**1.406**	**0.095**	**1.774**	**1.219**	**1.362**
salicylic acid	**1.466**	**1.410**	**0.056**	**1.336**	**1.220**	**1.338**
thiosalicylic acid	**1.483**	**1.411**	**0.072**	**1.767**	**1.212**	**1.345**
salicylamide	1.505	1.399	0.106	1.353	1.210	1.367
2-mercaptobenzamide	1.498	1.404	0.094	1.778	1.214	1.368
salicylic acid	1.485	1.406	0.079	1.346	1.202	1.356
thiosalicylic acid	1.480	1.407	0.073	1.774	1.206	1.347
Dimers						
benzamide	1.502	1.394	0.108		1.230	1.345
benzoic acid	1.486	1.394	0.092		1.224	1.313
salicylamide	**1.484**	**1.411**	**0.073**	**1.336**	**1.248**	**1.338**
2-mercaptobenzamide	**1.500**	**1.406**	**0.094**	**1.775**	**1.234**	**1.342**
salicylic acid	**1.465**	**1.411**	**0.054**	**1.336**	**1.240**	**1.309**
thiosalicylic acid	**1.483**	**1.411**	**0.072**	**1.768**	**1.230**	**1.314**
salicylamide	1.504	1.399	0.105	1.354	1.226	1.345
2-mercaptobenzamide	1.498	1.403	0.095	1.779	1.229	1.344
salicylic acid	1.486	1.406	0.080	1.348	1.220	1.319
thiosalicylic acid	1.480	1.407	0.073	1.774	1.225	1.314

a Results for systems with closed ring by intramolecular hydrogen bond (cis conformations) are bolded. Results for systems without intramolecular hydrogen bond (trans conformations) are presented in the normal mode. The Δ*r* parameter is included that is defined as the difference between bond lengths (C–C_ring_ – C=C_ring_).

Table 1 contains the Δ*r* parameter that is the difference between the C–C_ring_ and C=C_ring_ bond lengths.^53^ One can see that for the trans (open) systems Δ*r* amounts to ∼0.07–0.11 Å, both for monomers and for dimers. For the cis (closed) conformations, this difference is situated between 0.05 and 0.10 Å showing that the intramolecular hydrogen bonds enhance only slightly the π-electron delocalization that results in the greater equalization of bonds. This equalization is restrained by the relatively rigid benzene skeleton since one of carbon–carbon bonds considered here (C=C_ring_) forms a part of benzene ring. Thus, one can see that the processes typical for the so-called RAHB systems are limited for structures where one of double C=C bonds is replaced by the bond of the benzene-like system. The changes resulting from the closure of the six-atoms ring concern also the C–O/S_ring_ and C=O bonds. The C–O/S_ring_ bonds are shortened, while the C=O bonds are elongated. However, the elongation of the C=O bonds in dimers containing units with intramolecular hydrogen bonds results from both interactions of intra- and intermolecular hydrogen bonds. For example, for the monomer of salicylic acid, for the open conformation, the length of the C–O_ring_ bond is equal to 1.346 Å and it decreases to 1.336 Å for the closed conformation of monomer. For dimers of salicylic acid, the closure of the six atoms ring leads to the shortening of the C–O_ring_ bond from 1.348 to1.336 Å; these values are practically the same for the pair of corresponding monomers. In the case of C=O bonds their lengths for monomers (trans and cis conformations) and for dimers (trans and cis) are equal to 1.202 Å, 1.220 Å, 1.220, and 1.240 Å, respectively. It means that the C=O bond elongations resulting from the closure of the ring, i.e., the formation of intramolecular RAHB, and from the dimerization are approximately additive, and each effect is connected with the bond elongation by about ∼0.02 Å. Let us look at another four related species of 2-mercaptobenzamide (Scheme 2 shows the dimer cis conformation), and for the changes of the C=O bond length for the sequence of trans and cis monomers and trans and cis dimers, this length is equal to 1.214 Å, 1.219 Å, 1.229 Å, and 1.234 Å, respectively. It means that the elongation resulting from the ring closure is equal to 0.005 Å, while from the dimerization it is 0.015 Å; the closure and dimerization effects are additive.

Let us look at the changes of C–O/N_inter_ bonds which similar to C=O bonds are affected by both interactions: inter- and intramolecular RAHBs. These are the bonds adjacent to the N–H or O–H proton donating bonds of amide or carboxylic groups, respectively, in intermolecular RAHBs. The C–O/N_inter_ bond is shortened due to the formation of intermolecular RAHB, and the similar effect of shortening results from the formation of the intramolecular hydrogen bonds. The additivity of these effects is not so clear as in a case of the carbonyl C=O bonds discussed above here. However, one can observe that the geometrical changes resulting from intra- and from intermolecular hydrogen bonds are independent of one another. If one compares the C–C_ring_, C=C_ring_, and C–O/S_ring_ bonds (Table 1) of closed conformations in monomers with the corresponding bonds in dimer species, they are practically the same; differences between them do not exceed 0.002 Å. It means that the formation of the intermolecular RAHB does not affect the ring formed by intramolecular RAHB except for the C=O bond that participates in both interactions. The same concerns the trans conformations; the above-mentioned bond lengths are practically the same for monomers and for dimers.

Table 2 shows the geometrical parameters of the hydrogen bonds that occur in systems investigated here. One can perform the approximate comparison of the strength of these interactions since the hydrogen bond strength increases roughly with the shortening of the proton–proton acceptor distance.^3−5,50^ For example, for the intramolecular RAHBs, the shortest H···O distance is observed for the salicylamide, 1.698 Å and 1.691 Å for the monomer and the dimer, respectively. In the cis conformation of monomer it corresponds to the meaningful equalization of pairs of CC and CO bonds if they are compared with bonds of the monomer trans conformation. The small geometrical changes resulting from closure of *S*(6) rings accompany the structures of 2-mercaptobenzamide where the intramolecular H···O distances are equal to 2.00 and 2.02 Å for monomer and dimer, respectively. The shortest intermolecular H···O distances are observed for dimer of benzoic acid as well as for salicylic and thiosalicylic acids in dimers of both open and close conformations (Table 2). These short distances correspond to the greatest equalization of the C=O and C–O_inter_ bonds (Table 1)

**Table 2:** Geometrical Parameters (in Å) of the Intra- and Intermolecular Hydrogen Bonds^a^

	intramolecular (o-substituent)			intermolecular (COOH or CONH_2_ group)		
compd	O/S–H	H···O	O/S···O	O/N–H	H···O	O/N···O
Monomers						
benzamide				1.007		
benzoic acid				0.964		
salicylamide	**0.982**	**1.698**	**2.575**	**1.007**		
2-mercaptobenzamide	**1.346**	**2.000**	**3.107**	**1.007**		
salicylic acid	**0.975**	**1.772**	**2.628**	**0.964**		
thiosalicylic acid	**1.346**	**1.908**	**3.034**	**0.964**		
salicylamide	0.958		2.995	1.007		
2-mercaptobenzamide	1.346		2.833	1.007		
salicylic acid	0.959		2.698	0.963		
thiosalicylic acid	1.347		2.739	0.964		
Dimers						
benzamide				1.025	1.841	2.864
benzoic acid				0.994	1.659	2.653
salicylamide	**0.981**	**1.691**	**2.569**	**1.024**	**1.846**	**2.869**
2-mercaptobenzamide	**1.345**	**2.020**	**3.123**	**1.025**	**1.848**	**2.871**
salicylic acid	**0.974**	**1.766**	**2.620**	**0.993**	**1.667**	**2.660**
thiosalicylic acid	**1.343**	**1.914**	**3.030**	**0.991**	**1.674**	**2.665**
salicylamide	0.958		2.985	1.026	1.843	2.865
2-mercaptobenzamide	1.346		2.834	1.025	1.841	2.862
salicylic acid	0.959		2.677	0.994	1.661	2.665
thiosalicylic acid	1.347		2.715	0.993	1.664	2.657

a O–H or S–H or N–H bond lengths as well as the H···O and O···O or S···O or N···O distances are included. Results for systems with closed ring by intramolecular hydrogen bond are bolded. Results for systems without intramolecular hydrogen bond are presented in the normal mode.

The geometrical parameters of the intramolecular hydrogen bonds in monomers are very similar to those in dimers (Table 2). The same is observed for intermolecular hydrogen bonds; they differ only slightly for cis conformations and their trans counterparts. These differences are greater however than those observed for bonds between heavier atoms in the motifs corresponding to the hydrogen bonds (Table 1). This probably results from the H atom position in the hydrogen bond bridges that is more sensitive to the environmental effects than the positions of heavier atoms.

The lengths of proton donating bonds are also presented in Table 2. One can see that the complexation, i.e., the formation of hydrogen bonds between carboxylic or between amide groups in dimers, leads to the elongation of the O–H and N–H bonds by about 0.02–0.03 Å. It is known that the hydrogen bond formation is most often connected with the elongation of the proton donating bond. Such elongation was assumed in early studies as the signature of existence of this type of interaction.^25,26^ However, for some of the complexes the shortening of the proton donating bond is observed as a result of the hydrogen bond formation. This is accompanied by the shift of this bond stretching band toward higher frequencies.^54−57^ This is not the case for the intermolecular interactions analyzed here as well as for the O–H···O intramolecular hydrogen bonds where the elongation of the O–H proton donating bond by about ∼0.02 Å is observed. However, for the S–H···O intramolecular hydrogen bonds there is no significant change of the S–H bond length for 2-mercaptobenzamide and there is the slight bond shortening of 0.001 and 0.004 Å for thiosalicylic acid, for monomer and for dimer, respectively. The open (trans) conformations are reference systems for the O–H···O and S–H···O intramolecular hydrogen bonds discussed here. The shortenings of the S–H bond for thiosalicylic acid are accompanied by the blue shifts of the corresponding stretching modes by 1.5 and 16.6 cm^–1^ for the monomer and dimer, respectively (ωB97XD/6-311++G(d,p) level applied in this study). It is worth noting here that the blue-shifting intramolecular hydrogen bonds were analyzed in early studies and the problem of the choice of the reference systems was discussed there.^58,59^

### 3.3 QTAIM Characteristics of Inter- and Intramolecular Hydrogen Bonds

Table 3 presents selected QTAIM parameters of the inter- and intramolecular hydrogen bonds, the orbital–orbital interaction energies being characteristic for the hydrogen bond (eq 1) are also included (these energies are designated as Δ*E*_orb_). Several of interactions analyzed here are relatively strong, and they may be considered as partly covalent in nature. For such interactions the total electron energy density at the H···O bond critical point (BCP), *H*_BCP_, is negative.^50,60−62^ The negative value of the Laplacian of electron density at BCP, ▽^2^ρ_BCP_, is attributed to covalent bonds,^28^ and in the case of hydrogen bonds, i.e., BCPs of H···proton acceptor bond paths, it confirms the covalent character of this interaction.^50^ However, it seems that such a situation where ▽^2^ρ_BCP_ < 0 occurs only in a few cases of hydrogen bonds; (FHF)^−^ anion is an example.^50^ The systems characterized by partial covalency, with the negative *H*_BCP_ values, are more common. For the hydrogen bonds analyzed here, for all cases, ▽^2^ρ_BCP_ is positive but *H*_BCP_ is negative for all O–H···O arrangements, for intramolecular interactions of salicylamide and salicylic acid as well as for intermolecular interactions in benzoic acid, salicylic acid, and thiosalicylic acid. For these interactions the electron density at the H···O BCP, ρ_BCP_, is equal to or it exceeds 0.04 au. It is greater than the electron density at H···O BCP for the remaining intramolecular S–H···O and intermolecular N–H···O hydrogen bonds where the *H*_BCP_ value is positive. Such positive *H*_BCP_ values are observed in thiosalicylic acid as well as in benzamide, salicylamide, and 2-mercaptobenzamide. It is worth noting that the ρ_BCP_ value is often considered as a measure of the strength of interaction. The correlations between this QTAIM parameter and the other indicators of the strength of interaction, interaction energy, proton···proton acceptor distance, etc. were observed for numerous samples of hydrogen bonds.^50,63−69^

**Table 3:** QTAIM Parameters of Electron Density at BCP (ρ_BCP_) and Total Electron Energy Density at the BCP (*H*_BCP_) and NBO Energies (Δ*E*_orb_) Corresponding to the n_B_ → σ_AH_* Overlaps^a^

	cis conformations^b^					
	intramolecular HB O–H···O or S–H···O			intermolecular HB O–H···O or N–H···O		
compd	ρ_BCP_ (au)	*H*_BCP_ (au)	Δ*E*_orb_ (kcal/mol)	ρ_BCP_ (au)	*H*_BCP_ (au)	Δ*E* (kcal/mol)
benzamide				0.032	0.001	19.3
benzoic acid				0.048	–0.005	43.6
salicylamide	0.048	–0.005	26.0	0.031	0.001	16.8
	(0.048)	(−0.005)	(25.9)			
2-mercaptobenzamide	0.025	0.002	5.40	0.031	0.001	18.0
	(0.025)	(0.002)	(7.1)			
salicylic acid	0.040	–0.002	17.10	0.047	–0.005	32.1
	(0.040)	(−0.002)	(18.8)			
thiosalicylic acid	0.031	0.002	10.6	0.046	–0.004	30.7
	(0.031)	(0.002)	(12.9)			

	trans conformations					
	intramolecular ChB^c^S···O or O···O			intermolecular HB O–H···O or N–H···O		
compd	ρ_BCP_ (au)	*H*_BCP_ (au)	Δ*E*_orb_ (kcal/mol)	ρ_BCP_ (au)	*H*_BCP_ (au)	Δ*E* (kcal/mol)
salicylamide	O···O bond path not observed			0.032	0.001	19.2
2-mercaptobenzamide	0.017	0.001	2.0	0.032	0.001	18.6
	(0.016)	(0.001)	(2.3)			
salicylic acid	0.015	0.002	0	0.048	–0.005	35.2
	(0.014)	(0.002)	(0)			
thiosalicylic acid	0.021	0.001	1.6	0.047	–0.005	39.6
	(0.020)	(0.001)	(4.0)			

a The results for dimers as well as for monomers (in parentheses) are presented.

b Benzoic acid and benzamide are not classified as cis or trans conformations since o-substituents do not occur here.

c ChB: chalcogen bond.

The results presented in Table 3 confirm the conclusions of the former sections that the intramolecular and intermolecular hydrogen bonds are rather independent. The ρ_BCP_ values for intramolecular hydrogen bonds in monomers are the same as the values for the corresponding interactions in dimers! In the case of intermolecular hydrogen bonds the ρ_BCP_ values for the dimer closed (cis) conformations do not differ more than by 0.001 au from the corresponding values of the dimer open (trans) conformations. The NBO orbital–orbital overlap interaction energies, Δ*E*_orb_, seem to be more sensitive to the interrelation between intra- and intermolecular interactions. However, the energies related to the n_O_ → σ*_OH/SH_ overlaps for intramolecular hydrogen bonds are greater for the monomers than for dimers (except of salicylamide). It is reasonable since for dimers these energies are weaker because the carbonyl group oxygen participates in two interactions. In other words, bifurcated hydrogen bonds^70^ are observed here. Similarly the n_O_ → σ*_OH/NH_ overlaps related to the intermolecular interactions are stronger for the open conformations since the proton accepting oxygen centers do not participate in the additional intramolecular interactions. Thus, the NBO approach is more sensitive than other theoretical approaches to detect the interrelations between intra- and intermolecular interactions and particularly to detect the double role of the carbonyl oxygen center, as the proton acceptor in two hydrogen bonds.

**Figure 5:**
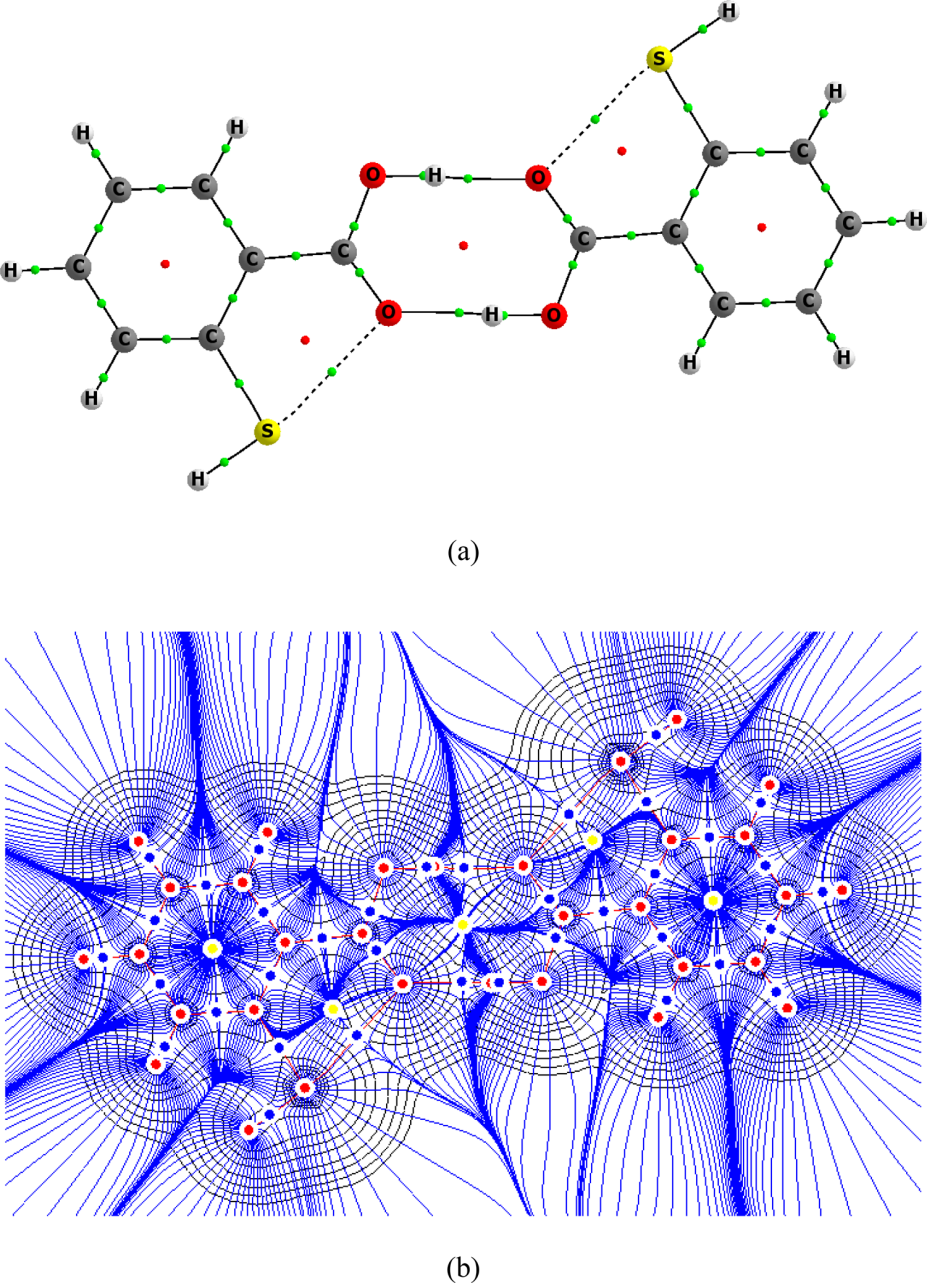
(a) Molecular graph of the dimer of the trans conformer of thiosalicylic acid, where big circles correspond to attractors, small ones to bond (green) and ring (red) critical points. (b) Molecular graph with electron density isolines (black) and the gradient paths (blue), where red lines are bond paths connecting attractors: red circles, attractors attributed to atoms; blue circles, bond critical points; yellow circles, ring critical points.

Table 3 shows the characteristics of BCPs of the S···O bond paths for the monomers and dimers of the trans conformations of 2-mercaptobenzamide and thiosalicylic acid as well as the characteristics for BCPs of the O···O bond paths of the trans conformations of monomer and dimer of salicylic acid. The classification of the O···O interactions as the stabilizing one seems to be problematic. For example, the meaning of the bond path was a subject of debates and controversies in general.^71−75^ However, the O···O interactions and particularly bond paths in dinitroamide anion and in other systems were discussed.^76^ In the case of salicylic acid are the low ρ_BCP_ values for O···O BCP observed here, lower than for the S···O links. Besides, for the O···O links and for monomer and for dimer open conformations, the overlaps corresponding to the stabilizing n_O_ → σ*_HO_ interactions are not observed. However, the n_O_ → σ*_HS_ overlaps are observed for the S···O links in thiosalicylic acid and 2-mercaptobenzamide with energies of interactions between 1.6 and 4.0 kcal/mol (Table 3). The ρ_BCP_ values for the S···O bond paths are situated between 0.016 and 0.021 au for these interactions which may be classified as chalcogen bonds.^46,77−80^Figure 5 presents the molecular graph of the thiosalicylic acid dimer where the S···O bond paths of the intramolecular chalcogen bonds are presented. The molecular graph with the isolines of the electron density and the gradient paths is also shown. It is worth noting here that these chalcogen bonds where the sulfur center acts as the Lewis acid may be classified as the σ-hole bonds according to the σ-hole concept.^77,81,82^

Hence one can see the variety of interactions presented in Table 3, similar to what was observed in former tables. If one assumes that the ρ_BCP_ value corresponds to the strength of the interaction, the following order is observed, starting from the strongest interactions: all inter- and intramolecular O–H···O hydrogen bonds (ρ_BCP_ values between 0.040 and 0.048 au), intermolecular N–H···O hydrogen bonds (ρ_BCP_ values between 0.031 and 0.032 au), intramolecular S–H···O interactions (ρ_BCP_ values of 0.025 and 0.031 au), and intramolecular chalcogen bonds (ρ_BCP_ values between 0.014 and 0.021 au).

### 3.4 SAPT Approach: The Nature of Intermolecular Hydrogen Bonds

The symmetry adapted perturbation theory (SAPT) approach^27^ was applied here to deepen the understanding of nature of O–H···O and N–H···O intermolecular hydrogen bonds in the analyzed dimers. Table 4 presents SAPT interaction energies, *E*_int_^SAPT2^, and their terms (see eq 2). The *E*_int_^SAPT2^ values between −12.6 kcal/mol and −17.5 kcal/mol are observed. However, it is worth recalling that they concern two equivalent hydrogen bonds which occur in all dimers analyzed here. The interactions are stronger for the dimers of open conformation, *E*_int_^SAPT2^, between −13.6 and −17.4 kcal/mol, than for the closed conformations, between −12.6 and −16.0 kcal/mol. The latter differences result from the bifurcation since the oxygen carbonyl atoms in closed conformations are involved in intra- and intermolecular interactions. The N–H···O hydrogen bonds are weaker than the O–H···O ones since the following ranges of the energy of interaction are observed; from −12.6 to −13.8 kcal/mol and from −15.7 to −17.5 kcal/mol, respectively. Table 4 shows that the SAPT interaction energy terms, up to the second order, cover the main part of the energy of interaction. However, the δ*E*_HF_ term is not negligible since it ranges from −3.8 to −7.4 kcal/mol.

**Table 4:** Interaction Energy Terms (in kcal/mol) That Are Defined by Equation 2^a^

compd	*E*_elst_^(1)^	*E*_exch_^(1)^	*E*_ind_^(2)^	*E*_disp_^(2)^	*E*_exch-ind_^(2)^	*E*_exch-disp_^(2)^	*δE*_HF_	*E*_int_^HF^	*E*_int_^SAPT2^
benzamide	–25.0	27.9	–13.7	–7.0	7.1	1.4	–4.1	–11.8	–13.4
benzoic acid	–34.2	42.9	–24.9	–9.0	13.1	1.9	–7.4	–16.5	–17.5
salicylamide, c	–23.4	26.4	–12.7	–6.9	6.5	1.3	–3.8	–11.2	–12.6
2-mercaptobenzamide, c	–23.8	26.9	–13.1	–7.1	6.9	1.3	–3.9	–11.1	–12.8
salicylic acid, c	–31.7	40.8	–23.6	–8.8	12.2	1.8	–6.9	–15.3	–16.0
thiosalicylic acid, c	–31.3	40.2	–23.1	–7.1	12.1	1.8	–6.7	–14.7	–15.7
salicylamide, o	–24.9	28.0	–13.9	–7.1	7.2	1.4	–4.2	–12.0	–13.6
2-mercaptobenzamide, o	–24.8	27.6	–13.6	–7.2	7.0	1.4	–4.1	–12.1	–13.8
salicylic acid, o	–34.0	42.8	–24.7	–9.1	13.0	2.0	–7.4	–16.6	–17.4
thiosalicylic acid, o	–33.1	41.7	–24.2	–7.2	12.5	1.9	–7.2	–16.5	–17.4

a *E*_int_^HF^ is the Hartree–Fock interaction energy. c marks the systems containing rings closed by intramolecular hydrogen bonds. o designates ortho-substituted “open” species containing trans arrangements.

Table 4 shows that for all dimers analyzed the electrostatic energy, *E*_elst_^(1)^, is the most important attractive interaction energy term followed by the induction energy, *E*_ind_^(2)^. For the dimers linked by the stronger O–H···O interactions the *E*_ind_^(2)^/*E*_elst_^(1)^ ratio is equal to 0.73–0.74, while for the dimers linked by the weaker N–H···O interactions this ratio amounts to 0.54–0.56. It means that with the increase of the total interaction strength the induction interaction energy increases more than the other attractive interaction energy terms. It was observed early for hydrogen bonds^83^ and for other types of interactions.^84^ The induction energy term is related to the electron charge shifts resulting from complexation. In various studies, it is often assigned to the covalent character of interaction. One can see that the SAPT results are in agreement with the QTAIM ones since for the O–H···O interactions the total electron energy density at BCP, *H*_BCP_, is negative while for the weaker N–H···O interactions the positive *H*_BCP_ values and the lower values of ρ_BCP_ are observed (see Table 3). The dispersion interaction energy, less important attractive interaction energy term than electrostatic and induction contributions, is not negligible, between −7 and −9 kcal/mol.

It is worth recalling that the interaction energy terms are not mutually independent^81,82^ and the separated absolute values of all interactions energy terms usually increase with the increase of the strength of interaction.^83,84^ Thus, the interaction energy terms often correlate with each other and with other parameters related to the strength of interaction. For the dimers analyzed here the correlation between the H···O intermolecular distance and the *E*_int_^SAPT2^ value is observed. The *R*^2^ for the linear relationship is equal to 0.994. The attraction follows the Pauli repulsion, and the former terms overwhelm the latter one. The linear correlation is also observed here, between the sum of attractive terms (*E*_elst_^(1)^ + *E*_ind_^(2)^ + *E*_disp_^(2)^) and the repulsion (*E*_exch_^(1)^) since *R*^2^ is equal to 0.996.

### 3.5 Electron Localization Function Approach

**Figure 6:**
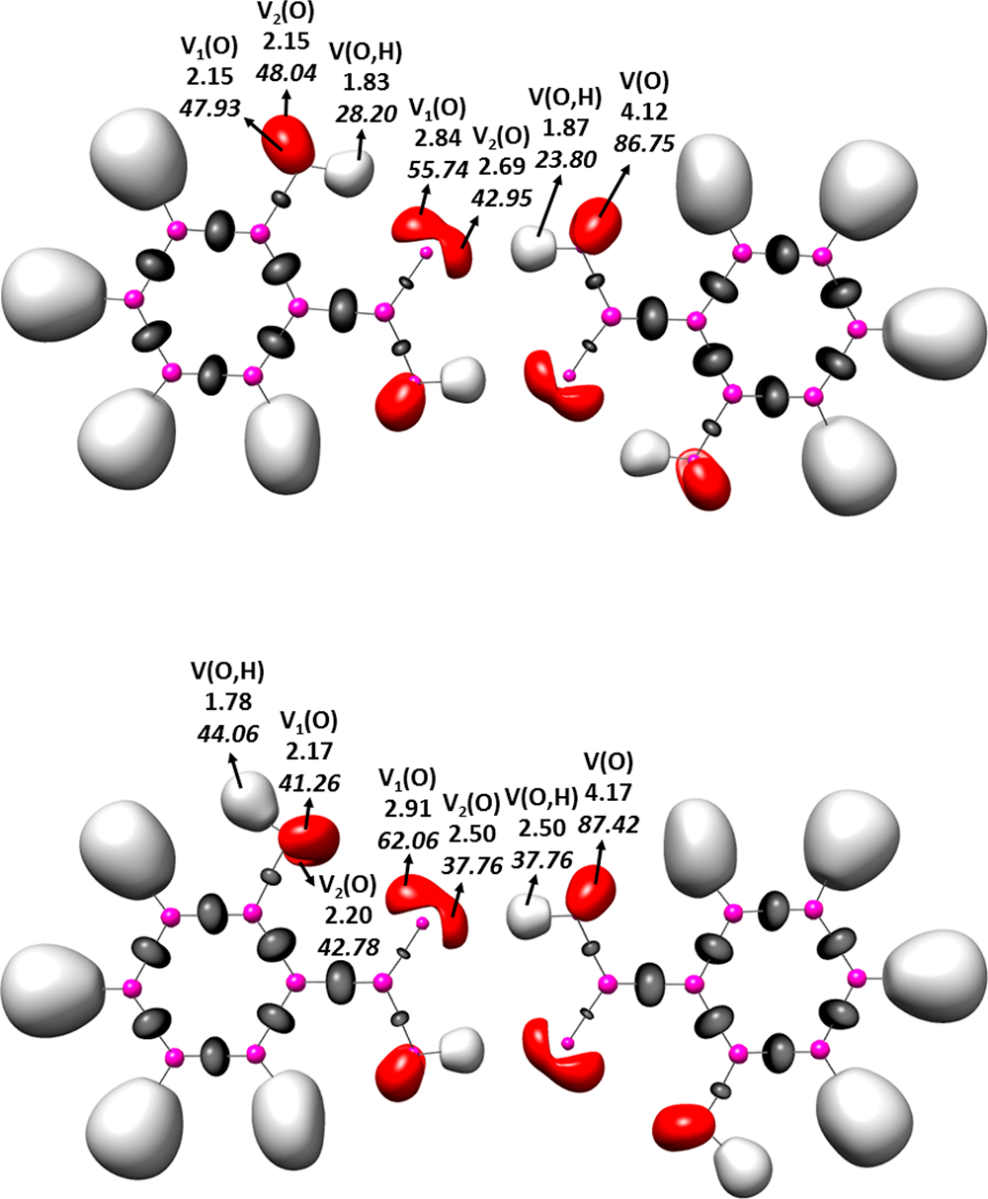
ELF isosurfaces (η(*r*) = 0.85) for the dimers of salicylic acid, for the cis conformation (up), and for the trans one (down).

The ELF calculations were performed for the monomers and dimers discussed here. Figure S1 (Supporting Information) presents isosurfaces of ELF function (η(*r*) = 0.85) for all systems analyzed. For selected basins related directly to hydrogen bonds the following characteristics are presented there: their populations, variances, volumes (in Å^3^), and ELF values. Figure 6 shows examples of two dimers of salicylic acid, for closed and for open conformations. Only basins populations and basis volumes are presented in this figure.

The choice of the ELF function value of 0.85 led to the partitioning of the molecular spaces where monosynaptic valence V(A) basins as well as the valence disynaptic protonated basins V(A,H) are observed. The basins related to hydrogen bonds are discussed here. Those related to other parts of molecules as well as other basins as for example core basins are not analyzed. For some of the atoms, the single monosynaptic valence basins corresponding to lone pairs occur. For the other ones the reduction of molecular space led to two valence basins.

Figure 6 (and Figure S1 for all species analyzed) do not show the monosynaptic basins V(H) which are the signature of very strong hydrogen bonds. This occurs in several complexes like, for example, for the FHF^–^ anion or for the H_5_O_2_^+^ cation.^85^Table 5 presents populations of the monosynaptic and disynaptic basins for the proton donating bonds. Their polarizations (POL%) calculated within the NBO approach are also included, which is shown later here that they are related to ELF approach parameters. This polarization in the NBO approach is understood as the percentage of electron density of the A–H proton donating bond at the A-center.^26^

**Table 5:** ELF Parameters^a^

	O/S–H o-substituent			O/N–H in COOH or CONH_2_		
compd	V(O/S,H)	V(O/S)	POL%	V(O/N,H)	V(O/N)	POL%
Monomers						
benzamide				1.99	1.39	70.24
benzoic acid				1.81	4.32	74.82
salicylamide	**1.84**	**4.29**	**77.47**	**1.98**	**1.26**	**70.46**
2-mercaptobenzamide	**1.87**	**4.25**	**59.70**	**1.99**	**1.28**	**70.37**
salicylic acid	**1.84**	**4.31**	**76.91**	**1.81**	**4.27**	**74.94**
thiosalicylic acid	**1.89**	**4.20**	**60.04**	**1.80**	**4.30**	**74.95**
salicylamide	1.78	4.41	73.81	1.99	1.29	70.20
2-mercaptobenzamide	1.87	4.24	55.21	1.99	1.40	70.27
salicylic acid	1.78	4.38	73.72	1.88	4.36	74.79
thiosalicylic acid	1.88	4.22	55.07	1.79	4.34	74.89
Dimers						
benzamide				2.06	1.00	73.53
benzoic acid				1.89	4.12	78.56
salicylamide	**1.85**	**4.29**	**77.39**	**2.05**	**0.89**	**73.46**
2-mercaptobenzamide	**1.87**	**4.26**	**59.37**	**2.07**	**0.92**	**73.42**
salicylic acid	**1.83**	**4.30**	**76.80**	**1.87**	**4.12**	**78.44**
thiosalicylic acid	**1.89**	**4.21**	**59.74**	**1.88**	**4.14**	**78.41**
salicylamide	1.77	4.43	73.76	2.06	0.92	73.49
2-mercaptobenzamide	1.87	4.24	55.28	2.06	1.00	73.53
salicylic acid	1.78	4.37	73.69	1.87	4.17	78.56
thiosalicylic acid	1.88	4.21	55.08	1.88	4.15	78.55

a Populations of the monosynaptic V(O), V(S), and V(N) as well as of disynaptic V(O,H), V(S,H), and V(N,H) valence basins of the proton donating bonds that may be involved in inter- and intramolecular interactions. The polarizations (POL%) of these bonds calculated within the NBO approach are included. Results for systems with closed ring by intramolecular hydrogen bond are bolded. Results for systems without intramolecular hydrogen bond are presented in the normal mode.

Let us consider parameters of the intramolecular hydrogen bonds, i.e., populations of disynaptic protonated V(O/S,H) basins, populations of monosynaptic V(O/S) basins, and the polarization of the proton donating O/S–H bonds (POL%) calculated within the NBO approach. If two V(O/S) valence basins occur, then the sum of their populations for the atom analyzed is included in Table 5. One can see that all parameters mentioned above of the O–H and S–H substituents differ only slightly, if any, for the monomers and for the corresponding dimers. It means that the intermolecular hydrogen bonds between carboxylic or amide groups do not affect the intramolecular systems. The latter conclusion is in force for the closed conformations where the intramolecular hydrogen bonds are observed and for the O–H or S–H bonds in the open conformers. It is in agreement with the observations of the former sections where it was found the intermolecular hydrogen bonds do not influence on the intramolecular arrangements. There are other interesting observations for the latter arrangements. The closure of the six-member rings, i.e., *S*(6) motifs, results in the increase of the V(O/S,H) populations and the decrease of the V(O/S) populations. However, these changes are meaningful for O–H···O hydrogen bonds, while they are very small or even negligible for the S–H···O systems. It may be explained in the following way. The former interactions are stronger than the latter ones, and for the former interactions the more pronounced electron density rearrangements are the result of the hydrogen bond formation occuring compared with the latter ones. The polarization of the O/S–H proton donating bonds (Table 5, POL% values) increases if the hydrogen bonds are formed. However, this increase in the closed systems, in comparison to the corresponding open ones, is approximately the same for both types of interactions O–H···O and S–H···O, which is about ∼4%.

Let us analyze the intermolecular interactions. If one compares the parameters of monomers of the O–H or N–H proton donating bonds in carboxylic or amide groups, respectively, with the corresponding parameters in dimers, then one can see the similar changes as those that occur for the formation of intramolecular hydrogen bonds. The formation of intermolecular O–H···O and N–H···O intermolecular hydrogen bonds leads to the increase of the V(O/N,H) populations and the decrease of the V(O/N) populations. The corresponding increase of the POL% values by about 3.5–4% is observed. It is a little greater for the stronger O–H···O hydrogen bonds than for the weaker N–H···O interactions. The increase of the polarization of the proton donating bond that results from the hydrogen bond formation is an effect related to the electron charge shifts. This shift from the Lewis base center to the A–H proton donating bond occurs and later the inner shift within this bond from the H-atom to the more electronegative A-center, which leads to the increase of the bond polarization.^25,26^

If one compares for the intermolecular hydrogen bonds the ELF and NBO parameters discussed above for the trans and cis conformations, there is no meaningful differences between them. Such differences practically do not occur for V(O/N,H) populations, and they are small for V(O/N) ones. For the POL% values the differences are usually lower than 0.1–0.2%. These results show that there is no influence between the intermolecular and intramolecular interactions for the systems analyzed here.

## 4 Summary and Conclusions

The monomers and dimers of benzoic acid and benzamide as well as of their o-substituted derivatives were analyzed. The searches performed through the Cambridge Structural Database show that the majority of o-substituted species is characterized by the intramolecular hydrogen bonds where the carbonyl oxygen of the carboxylic or amide group plays a role of the Lewis base center. The DFT calculations performed on dimers of salicylic acid and salicylamide as well as on their sulfur counterparts confirm the CSD searches. They show that the centrosymmetric dimers linked by two equivalent intermolecular hydrogen bonds that also contain intramolecular hydrogen bonds with the carbonyl oxygen proton acceptor possess the lowest energies in comparison with other conformations. This is the reason for the most frequent occurrence of such systems in crystal structures.

The results of calculations also show that for dimers containing the S–H proton donating bond in the ortho position the energy differences between conformers are not as large as for o-substituents containing the O–H bond. This is the reason why in the crystal structure of thiosalicylic acid the centrosymmetric dimers linked by the O–H···O are observed that are stabilized by the intramolecular S···O chalcogen bonds. The evidence of the existence of the latter interactions for trans conformations of thiosalicylic acid and 2-mercaptobenzamide, for monomers and for dimers, is confirmed by the QTAIM and NBO approaches.

All theoretical results show that the intramolecular and intermolecular hydrogen bonds observed for the systems analyzed here are independent; rather, it means that they do not influence each other. This is confirmed by geometrical and topological (QTAIM and ELF) results. However, the slight differences in energetic parameters indicate some slight reciprocal influence of these interactions; this is indicated by the results of NBO and SAPT approaches. The intermolecular interactions in dimers of open (trans) conformations are stronger than the intermolecular interactions in dimers of closed (cis) conformations. Similarly the orbital–orbital overlap interactions for intramolecular hydrogen bonds are stronger for monomers rather than for dimers where additional intermolecular hydrogen bonds are observed. The bifurcation effect occurs in dimers because the carbonyl oxygen participates in inter- and intramolecular hydrogen bonds.

The QTAIM approach and the energetic results, among them SAPT results, show that all O–H···O hydrogen bonds may be classified as partially covalent in nature interactions. The weaker N–H···O and S–H···O hydrogen bonds are mainly electrostatic in nature. The ELF results are in agreement with the NBO findings concerning the increase of polarization of the proton donating bonds resulting from the hydrogen bond formation. This increase is much greater for the stronger O–H···O hydrogen bonds than for the N–H···O and S–H···O interactions. The ELF approach provides parameters describing the electron charge redistribution resulting from the hydrogen bond formation, among them populations of the monosynaptic valence V(A) basins and the valence disynaptic protonated basins V(A,H) of the proton donating A–H bond.

## Supporting Information Available

ELF isosurfaces (η(*r*) = 0.85) for the monomers and dimers analyzed in this study, showing monosynaptic and disynaptic basins resulting from the molecular space reduction and including the following characteristics: the basinś populations, variances, volumes, and ELF values (PDF)
