# Supplementary material for: Coexistence of Intra- and Intermolecular Hydrogen Bonds: Salicylic Acid and Salicylamide and Their Thiol Counterparts
Source: J Phys Chem A. 2021 Feb 16;125(7):1526–39. doi: 10.1021/acs.jpca.0c11183 (PMC9171818; doi:10.1021/acs.jpca.0c11183)
Supplement: Supplementary file 1 — jp0c11183_si_001.pdf [file jp0c11183_si_001.pdf]

Supporting Information

Coexistence of Intra- and Intermolecular Hydrogen Bonds: Salicylic Acid and Salicylamide and their Thiol Counterparts

by

Samira Gholami, Mohammad Aarabi and Sławomir J. Grabowski *

Corresponding Author

Sławomir J. Grabowski - Faculty of Chemistry, University of the Basque Country and Donostia International Physics Center (DIPC), P.K. 1072, 20080 Donostia, Spain

IKERBASQUE, Basque Foundation for Science, 48011 Bilbao, Spain

orcid.org/0000-0003-4161-2938

e-mail: s.grabowski@ikerbasque.org

Authors

Samira Gholami - Dipartimento di Chimica Industriale, Università degli Studi di Bologna, Viale del Risorgimento 4, I-40136 Bologna, Italy

Mohammad Aarabi - Dipartimento di Chimica Industriale, Università degli Studi di Bologna, Viale del Risorgimento 4, I-40136 Bologna, Italy

Fig. S1 The ELF isosurfaces (η(r)=0.85) for the monomers and dimers analysed in this study; the monosynaptic and disynaptic basins resulting from the molecular space reduction are presented; the cis (closed) and trans (open) conformations are considered. The following characteristics are presented: the basins´ populations, variances, volumes (in Å^3) and ELF values that is shown for the first picture.

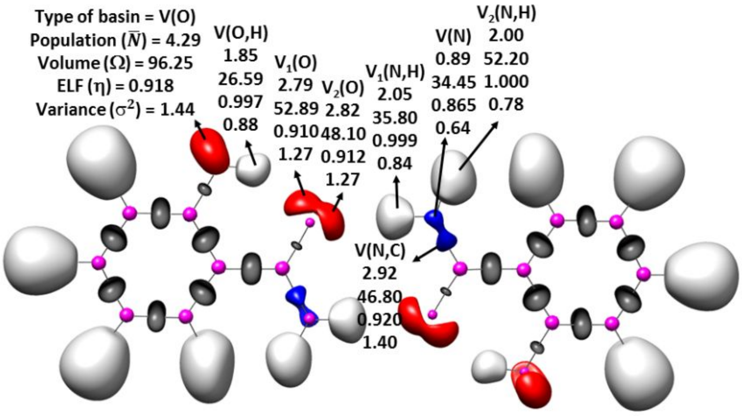

Salicylamide, dimer, closed conformation

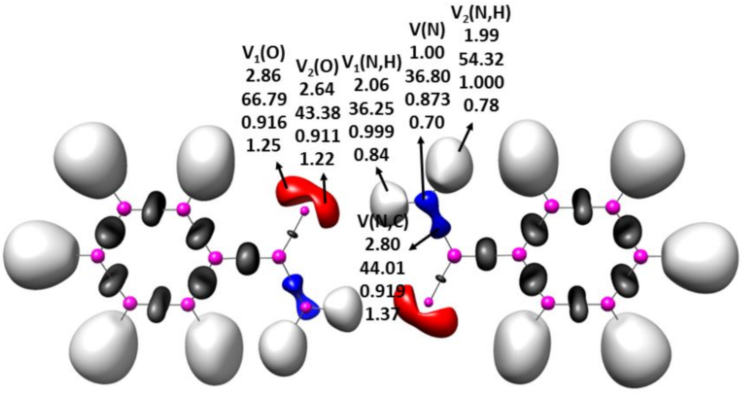

Benzamide, dimer

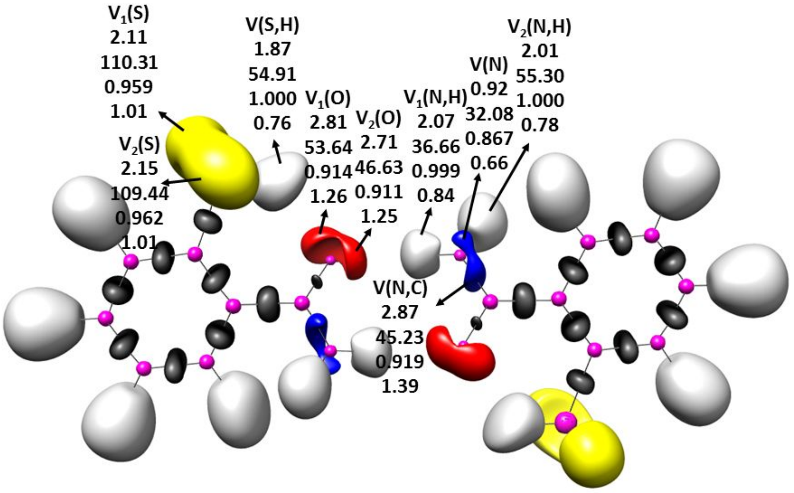

2-mercaptobenzamide, dimer, closed conformation

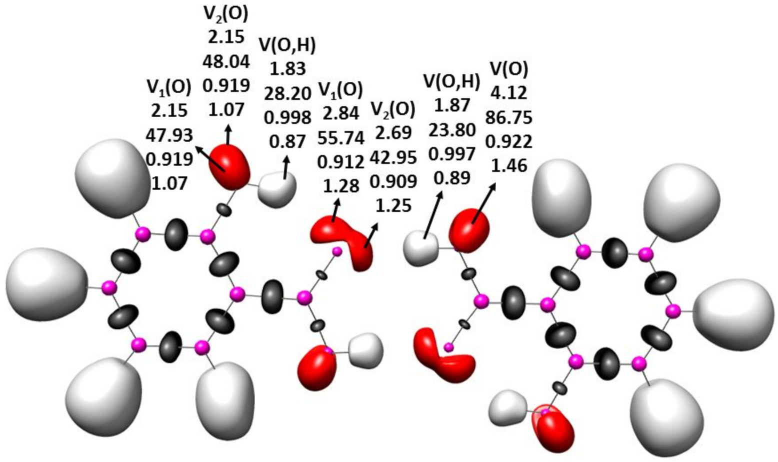

Salicylic acid, dimer, closed conformation

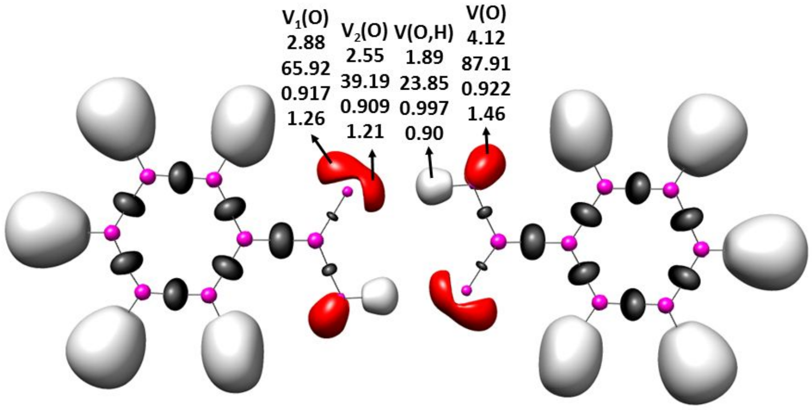

Benzoic acid, dimer

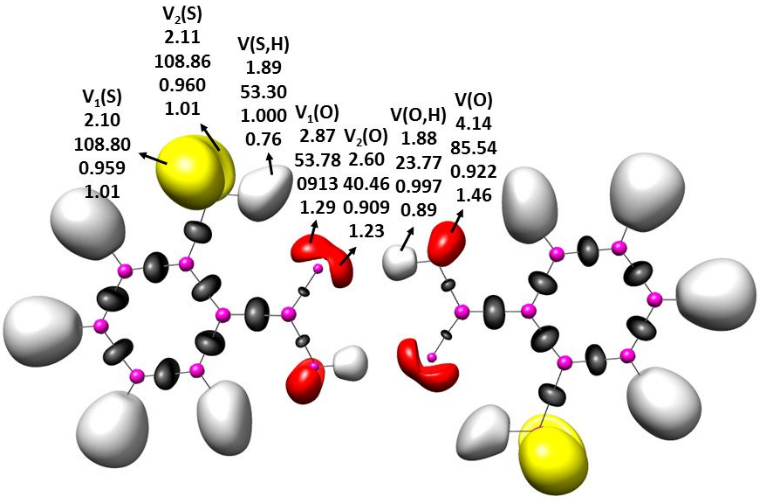

Thiosalicylic acid, dimer, closed conformation

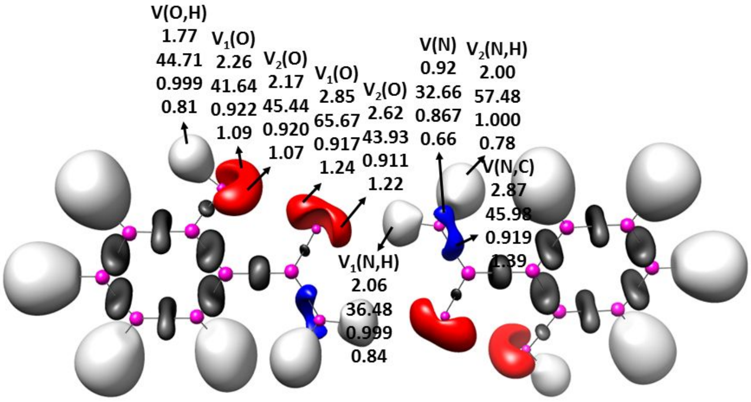

Salicylamide, dimer, open conformation

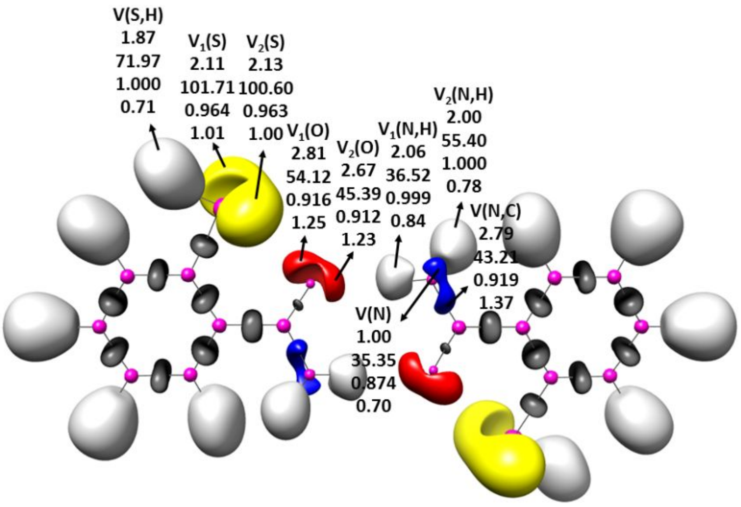

2-mercaptobenzamide, dimer, open conformation

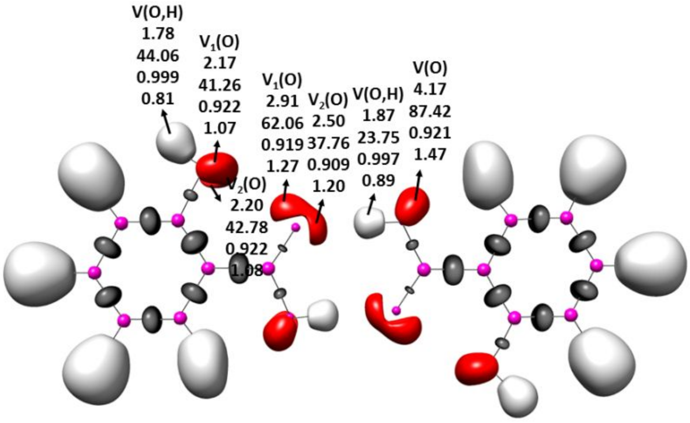

Salicylic acid, dimer, open conformation

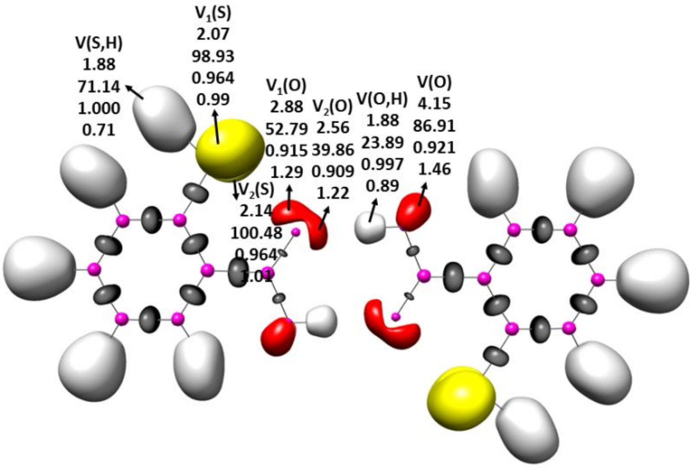

Thiosalicylic acid, dimer, open conformation

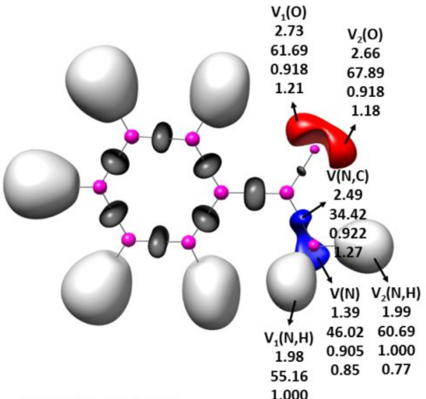

Benzamide, monomer

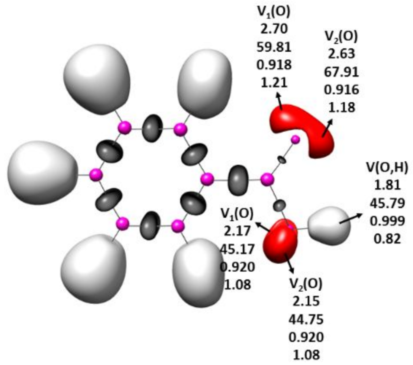

Benzoic acid, monomer

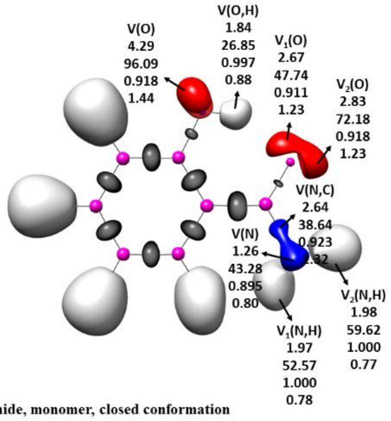

Salicylamide, monomer, closed conformation

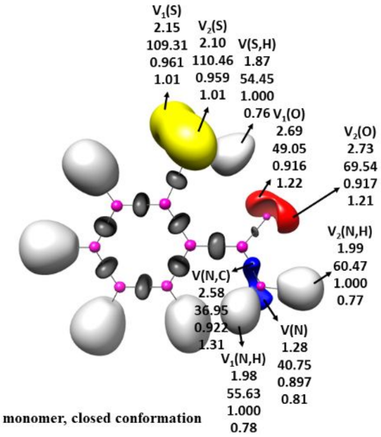

2-mercaptobenzamide, monomer, closed conformation

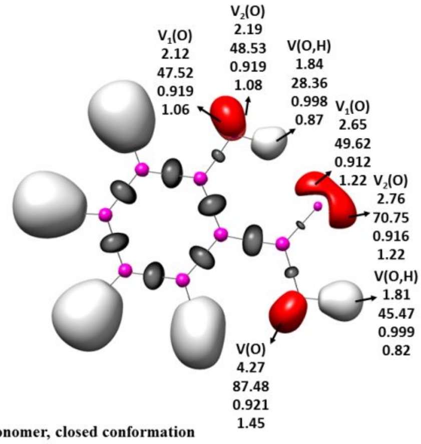

Salicylic acid, monomer, closed conformation

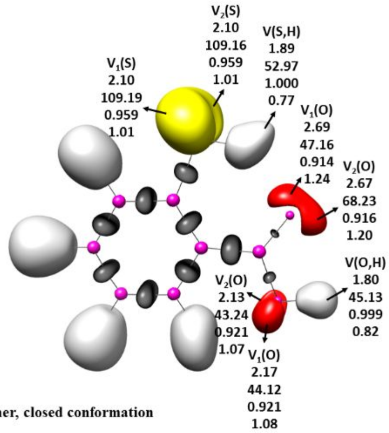

Thiosalicylic acid, monomer, closed conformation

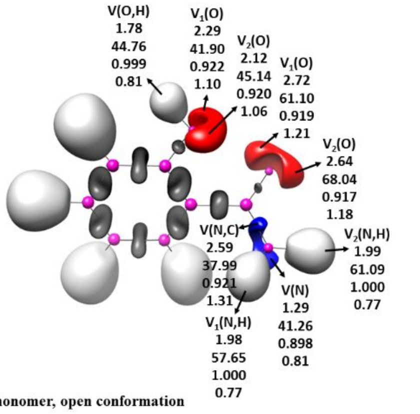

Salicylamide, monomer, open conformation

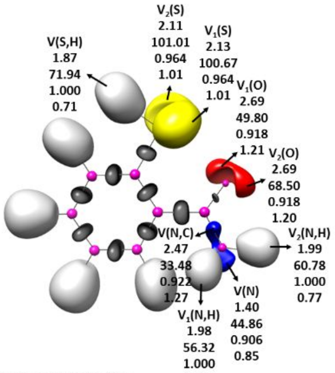

2-mercaptobenzamide, monomer, open conformation

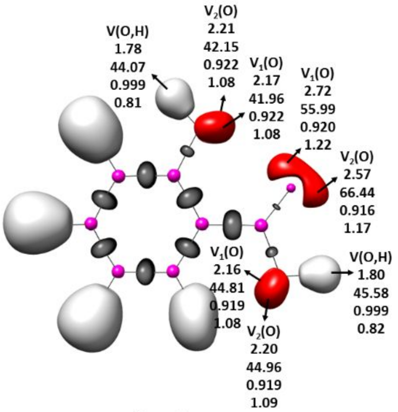

Salicylic acid, monomer, open conformation

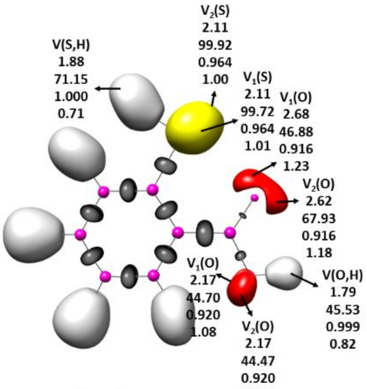

Thiosalicylic acid, monomer, open conformation
